# Mineralocorticoid Receptor and Sleep Quality in Chronic Kidney Disease

**DOI:** 10.3390/ijms252212320

**Published:** 2024-11-16

**Authors:** Juan de la Puente-Aldea, Oscar Lopez-Llanos, Daniel Horrillo, Hortensia Marcos-Sanchez, Sandra Sanz-Ballesteros, Raquel Franco, Frederic Jaisser, Laura Senovilla, Roberto Palacios-Ramirez

**Affiliations:** 1 Unidad de Excelencia Instituto de Biología y Genética Molecular (IBGM), Universidad de Valladolid—CSIC, 47003 Valladolid, Spain; jpualdea@gmail.com (J.d.l.P.-A.); oscarlopezllanos@gmail.com (O.L.-L.); laurasenovilla@hotmail.com (L.S.); 2 Facultad de ciencias de la Salud, Universidad Rey Juan Carlos, 28922 Alcorcon, Spain; danielhorrillo@gmail.com (D.H.); raquel.franco@urjc.es (R.F.); 3 Hospital Clínico Universitario, 47003 Valladolid, Spain; hmarcoss@saludcastillayleon.es (H.M.-S.); ssanzb@saludcastillayleon.es (S.S.-B.); 4 INSERM U1166, Team Diabetes, Metabolic Diseases and Comorbidities, Sorbonne Université, 75013 Paris, France; frederic.jaisser@gmail.com; 5 INSERM UMR 1116, Centre d’Investigations Cliniques-Plurithématique 1433, Université de Lorraine, CHRU de Nancy, French-Clinical Research Infrastructure Network (F-CRIN) INI-CRCT, 54500 Nancy, France; 6 INSERM U1138, Centre de Recherche des Cordeliers, Equipe Labellisée par la Ligue Contre le Cancer, Sorbonne Université, Institut Universitaire de France, 75006 Paris, France; 7 Metabolomics and Cell Biology Platforms, Institut Gustave Roussy, 94805 Villejuif, France

**Keywords:** mineralocorticoid receptor, sleep quality, circadian clock, chronic kidney disease

## Abstract

The classical function of the mineralocorticoid receptor (MR) is to maintain electrolytic homeostasis and control extracellular volume and blood pressure. The MR is expressed in the central nervous system (CNS) and is involved in the regulation of the hypothalamic–pituitary–adrenal (HPA) axis as well as sleep physiology, playing a role in the non-rapid eye movement (NREM) phase of sleep. Some patients with psychiatric disorders have very poor sleep quality, and a relationship between MR dysregulation and this disorder has been found in them. In addition, the MR is involved in the regulation of the renal peripheral clock. One of the most common comorbidities observed in patients with chronic kidney disease (CKD) is poor sleep quality. Patients with CKD experience sleep disturbances, including reduced sleep duration, sleep fragmentation, and insomnia. To date, no studies have specifically investigated the relationship between MR activation and CKD-associated sleep disturbances. However, in this review, we analyzed the environment that occurs in CKD and proposed two MR-related mechanisms that may be responsible for these sleep disturbances: the circadian clock disruption and the high levels of MR agonist observed in CKD.

## 1. Introduction

Activation of the mineralocorticoid receptor (MR) and its role in the hypothalamic–pituitary–adrenal (HPA) axis have been implicated in sleep physiology. Activation of the MR is a necessary condition to establish and maintain a good night’s sleep, especially in the non-rapid eye movement stage [1]. In addition, the MR has been studied in sleep deregulation, especially in pathological contexts such as major depression disorder (MDD) or autism spectrum disorder (ASD). In all these pathologies, MR under/overactivation has been associated with refractivity to treatment and a worse prognosis and worse symptoms, including sleep disturbances [2,3]. Sleep disorders, including restless legs syndrome (RLS), sleep apnea, poor sleep quality, and insomnia, are a common comorbid condition observed in individuals with chronic kidney disease (CKD) [4]. These disorders have a significant negative impact on the quality of life of CKD patients, with insomnia being up to three times more prevalent in patients with CKD than in the general population. Furthermore, this prevalence appears to be positively correlated with CKD progression and patient age [5]. The role of the MR in the pathophysiology of CKD is well established. MR antagonists (MRAs), including both classical steroidal and modern non-steroidal MRAs, have been shown to halt CKD progression and improve outcomes in comorbidities such as cardiovascular and metabolic diseases [6,7,8]. Despite significant advances in our understanding of sleep processes, the phenomenon of sleep remains largely enigmatic. Given the involvement of the MR in both CKD and sleep physiology, this review discusses what role the MR might play in CKD-associated sleep disturbances. In addition, we propose two MR-related mechanisms that could be responsible for these CKD-related sleep disturbances.

## 2. Mineralocorticoid Receptor

The nuclear receptor subfamily 3 group C member 2 (*NR3C2*) gene encodes the protein known as the MR. The MR is located in humans at cytogenetic position 4q31.23 and is approximately 363 kb with 13 exons (NCBI gene ID: 4306). This receptor belongs to the steroid hormone receptor family and can be activated by mineralocorticoids and glucocorticoids with similar affinity, although its canonical ligand is the mineralocorticoid aldosterone [9]. The MR has three functional domains: an N-terminal domain (NTD) that interacts with the coregulatory protein, a DNA-binding domain that binds to target genes in hormone response elements (HREs) and assists in dimerization, and a C-terminal ligand-biding domain (LBD) that interacts with chaperones in the absence of a ligand and allows the conformational change [9,10]. In the absence of a ligand, the MR remains in the cytoplasm in association with cytoplasmic proteins, such as heat shock protein (HSP) 90, HSP70, p23, FK506-binding protein (FKBP)51, FKBP52, cyclophilins, or serine/protein phosphatase 5, to maintain the receptor conformation for ligand-specific binding and avoid non-specific activation. Ligand binding induces a ligand-specific conformational change, allowing nuclear translocation facilitated by HSP90 and FKBP51, dimerization with another MR or glucocorticoid receptor (GR), post-translational modifications, and interaction with DNA and coregulatory proteins. The MR can act as a transcription factor by binding to HREs or by interacting with the epidermal growth factor receptor (EGFR). Once the MR is associated with HREs, it interacts with certain coactivators to enhance transcription, such as steroid receptor coactivator 1 (SRC-1), SRC-2, peroxisome proliferator-activated receptor γ coactivator 1-α, or the histone acetyltransferase p300/CREB-binding protein, among others [9].

### 2.1. Mineralocorticoid Receptor Ligands

The MR can bind with high affinity to several types of steroids, including aldosterone, deoxycorticosterone, glucocorticoids (cortisol, corticosterone in rodents) and progesterone. However, in the case of progesterone, even when it binds the MR with high affinity, it has a predominant anti-mineralocorticoid effect [11]. Aldosterone is considered the canonical ligand of the MR under physiological conditions [12]. The circulating concentration of cortisol is 100–1000 times higher than that of aldosterone and can bind to the MR with similar affinity [9,13].

Many of the corticosteroids listed in Table 1 have important pharmacological uses. Glucocorticoids, such as dexamethasone, are used as anti-inflammatory and immunosuppressive agents for the treatment of many diseases [14]. In addition, their role in wound healing has been demonstrated [15], as well as their usefulness in the treatment of various ocular diseases [16]. Mineralocorticoid antagonists have been proposed as potential key drugs in the treatment of various renal pathologies, including CKD, due to their ability to modulate stress activation systems [17]. On the other hand, some MR agonists, such as fludrocortisone (9a-fluorocortisol), have shown their efficacy in the treatment of hypotension [18]. Likewise, their combination with hydrocortisone has been proven to be effective in the treatment of septic shock [19]. Although known to be a weak partial agonist and a competitive MRA [20,21], progesterone binds to the MR with high affinity in vitro [22], and its binding affinity in vivo is even significantly higher than that of aldosterone [23,24,25].

The mechanisms by which the MR achieves differential activation by the two corticosteroid hormones and exerts tissue-specific effects can be considered at the pre-receptor, receptor, and post-receptor levels. Pre-receptor metabolism of cortisol is a key mechanism for maintaining MR selectivity in specific target tissues. 

The activity of 11β-hydroxysteroid dehydrogenase type 2 (11β-HSD2) converts cortisol in humans and corticosterone in mice to its inactive forms, cortisone and 11-dehydrocorticosterone, respectively [49]. However, this mechanism is only present in epithelial cells of the distal nephron, colon, sweat glands, and blood vessel walls [50]. Expression of 11β-HSD2 is minimal or absent in other MR-expressing tissues, such as the myocardium and hippocampus. At the receptor level, mineralocorticoid selectivity is produced by ligand-induced conformational changes that differ between cortisol and aldosterone. It has been shown that aldosterone dissociates more slowly from the MR and induces greater transactivation than cortisol at any given concentration [51]. Furthermore, the interaction between the NTD of the MR and the C-terminal hinge and LBD (or N-/C-interaction), which may serve to stabilize ligand binding to the receptor [52], is much stronger in the presence of aldosterone than cortisol, providing another mechanism of ligand specificity in the MR [53]. At the post-receptor level, an expanding library of more than 300 nuclear receptor (NR) co-regulators have been identified in the last decade [54]. NR co-regulators play a central role in the modulation of NR-mediated gene expression and are thought to confer tissue and ligand specificity due to their structural and functional diversity [55].

Receptor isoforms generate diversity in the tissue-specific receptor response. This has been demonstrated in the case of the GR, which has multiple potential isoforms, derived from the same gene, capable of eliciting unique biological responses to the same hormone in different tissues [56]. Transcription of the MR can be directed by two functional promoters, P1 and P2, to generate human MR-α and human MR-β isoforms that differ in the 5′ untranslated region and show differential tissue expressions [57]. Furthermore, both promoters are found to be glucocorticoid-inducible by glucocorticoids, whereas only P2 appears to be sensitive to mineralocorticoids, suggesting that ligand specificity may also be determined by the MR isoform generated.

### 2.2. Canonical Function of the Mineralocorticoid Receptor 

The associated classical function of the MR encompasses electrolytic homeostasis, extracellular volume control, and blood pressure regulation [10,58]. Eight days after birth, genetic inactivation of the MR in mice triggers hyperkalemia, hyponatremia, a sharp increase in plasma renin, angiotensin II, and aldosterone, and a potent reduction in epithelial sodium channel (ENaC) levels, although its mRNA levels are unchanged. These mice die within 10 days of birth due to water and Na^+^ loss [59].

In general terms, MR activation leads to Na^+^ retention and K^+^ secretion at the distal nephron [59]. Na^+^ levels are mainly controlled by the MR through the Na^+^−Cl^−^ cotransporter (NCC) in the early and late distal convoluted tube (DCT) and the ENaC present from the late DCT to the cortical collecting duct (CCD), which allows Na^+^ entry into the apical membrane of epithelial cells [60]. Na^+^ entry in these cells is due to the presence of Na^+^/K^+^ ATPase in the basolateral region, keeping intracellular Na^+^ low [59,60]. Na^+^ uptake by the ENaC depolarizes the apical membrane, but at the same time, the renal outer medullary K^+^ channel (ROMK) and Ca^2+^−regulated K^+^ channels (BK or Maxi-K) or the KCl cotransporter allows K^+^ secretion to the lumen under normal conditions. When K^+^ levels are too low, a change in K+ flux can occur, resulting in K^+^ uptake through the apical H^+^/K^+^ ATPase pump [10,59,60]. Upstream of the ENaC is the NCC, which allows reabsorption of Na^+^ and Cl^−^ without the need to excrete K^+^. Its inhibition permits the delivery of Na^+^ to the ENaC, although this does not stimulate K^+^ secretion [60]. Although the NCC is mainly regulated by peritubular K^+^ levels [10], it has been reported that chronic stimulation with aldosterone increases NCC expression in the distal nephron [10]. The MR response promotes the increase in ENaC and Na^+^/K^+^ ATPase activity, whereas the late response promotes the upregulation of these channels [10].

Electrolytic homeostasis is largely regulated by the MR through several molecular mechanisms including transcriptional or non-genomic regulation. First, the MR acts as a transcriptional factor leading to the generation of proteins such as period homolog 1 (PER1), endothelin 1 (ET-1), serum- and glucocorticoid-regulated kinase 1 (SGK1), glucocorticoid-induced leucine zipper, or ENaC [10]. Second, the MR also acts through the EGFR or insulin-like growth factor [10].

Upon activation, the MR triggers a quick increase in SGK1 expression. SGK1 is a major kinase for the MR to exert its function in tubular channels [60,61]. In addition, SGK1 phosphorylates Lysine Deficient Protein Kinase 4, preventing the endocytosis of the ENaC and ROMK and leading to the α subunit of the ENaC increasing its electrophysiological function [61]. Additionally, SGK1 phosphorylates ROMK, increasing its intracellular traffic to enhance its presence in the membrane, and participates in the activation of BK channels and other Ca^2+^, Mg^2+^, and Cl^−^ channels [61]. 

Moreover, MR action leads to an indirect increase in blood volume and blood pressure. Blood pressure depends on cardiac output and vascular resistance. At the same time, cardiac output depends on the stroke volume, which is modified by changes in blood volume and heart rate [62,63]. Na^+^ is the main osmolyte in the extracellular space and it moves parallel to water, which means that the accumulation of Na^+^ in the body increases blood volume and, therefore, blood pressure [63]. The kidney eliminates excess Na^+^ by increasing diuresis. To eliminate Na^+^ without losing water, the kidney uses urea as an osmolyte, although the total balance also depends on metabolic, respiratory, fecal, and transcutaneous water. The objective of the kidney is to maintain blood Na^+^ concentration, but the amount of Na^+^ in the tissues depends on blood Na^+^ and this is variable [63].

### 2.3. Non-Canonical Functions of the Mineralocorticoid Receptor 

The MR also has other non-canonical functions, such as the control of arterial pressure through the MR present in endothelial cells and vascular smooth muscle cells (VSMC). The endothelial MR does not affect blood pressure under basal conditions. However, in pathological conditions or conditions of MR overexpression, its activation modifies arterial pressure and vascular status by regulating vascular reactivity [62,64]. Endothelial MR activation leads to exocytosis of the Weibel–Palade body containing proinflammatory cytokines, an increase in reactive oxygen species by stimulation of nicotinamide adenine dinucleotide phosphate oxidase, and a reduction in glucose 6 phosphate dehydrogenase. These changes reduce nitric oxide (NO) availability, leading to endothelial dysfunction and even prothrombotic effects when the endothelium is injured. On the other hand, the MR of VSMCs regulates vascular tone by promoting the expression of L-type Ca^2+^ channels, which potentiate the contractile response and promote vascular remodeling and calcification, increasing vascular stiffness [58,62,64].

Many pathological processes are associated with chronic or increased activation of the MR. Continued activation of the MR can lead to fluid retention due to excessive NaCl reabsorption resulting in hypertension, alkalosis due to NaHCO_3_ retention, and proteinuria [10,64]. Sustained MR activation is also associated with fibrosis, extracellular matrix remodeling, and cell proliferation. In the cardiac context, it has been associated with atrial fibrillation or ventricular arrhythmias [58]. In the context of aging in mice, the MR of macrophages plays a proinflammatory and fibrogenic role [65,66]. Adipocytes also present the MR, which is involved in adipocyte differentiation and secretion of adipokines and proinflammatory markers implicated in insulin resistance and adipocyte dysfunction. In the retina, the MR maintains water and K^+^ homeostasis, leading to chronic aldosterone infusion, retinal ganglion cell death, and optic nerve degeneration [58].

### 2.4. The Mineralocorticoid Receptor in the Central Nervous System

Corticosteroids produce diverse actions in many organs and in various areas of the brain through the activation of the MR and GR. Both receptors show a specific and selective distribution in the brain [67,68]. The MR is strongly expressed in the hippocampus and medial and central amygdala, olfactory nucleus, layer II of the cortex, and motor and brainstem neurons. Expression is low in the hypothalamus and is found mainly in the paraventricular nucleus (PVN). In contrast, the GR is more ubiquitously expressed, with elevated expression found in the hippocampus, septum, hypothalamus, amygdala, and cerebral cortex [69]. Of note, the brain regions where these receptors are expressed are important for declarative and spatial memory. Moreover, the region in the amygdala with the MR and GR is important for emotional memory. Therefore, these receptors play a key role in the function and effect of stress, memory consolidation, and learning [70,71]. 

The MR is also expressed in neurons in brain areas of the autonomous nervous system [69]. In neurons belonging to the sympathetic nervous system, the function of aldosterone/MR activation is the regulation of cardiac activity and blood pressure by sympathetic activation [72,73]. However, as explained above, the MR also binds cortisol with a higher affinity than aldosterone. In some areas of the organism, such as the kidney, this binding is prevented by 11β-HSD2, which converts cortisol to cortisone. This does not usually occur in the central nervous system (CNS), which allows cortisol to bind to the MR [74]. Nevertheless, there are sites of aldosterone-preferential MR activation in the CNS, areas where the MR is co-expressed with 11β-HSD2, and which are involved in salt homeostasis, volume regulation, and sympathetic outflow. These areas are in the anterior hypothalamus and circumventricular organs [75].

**Figure 1:**
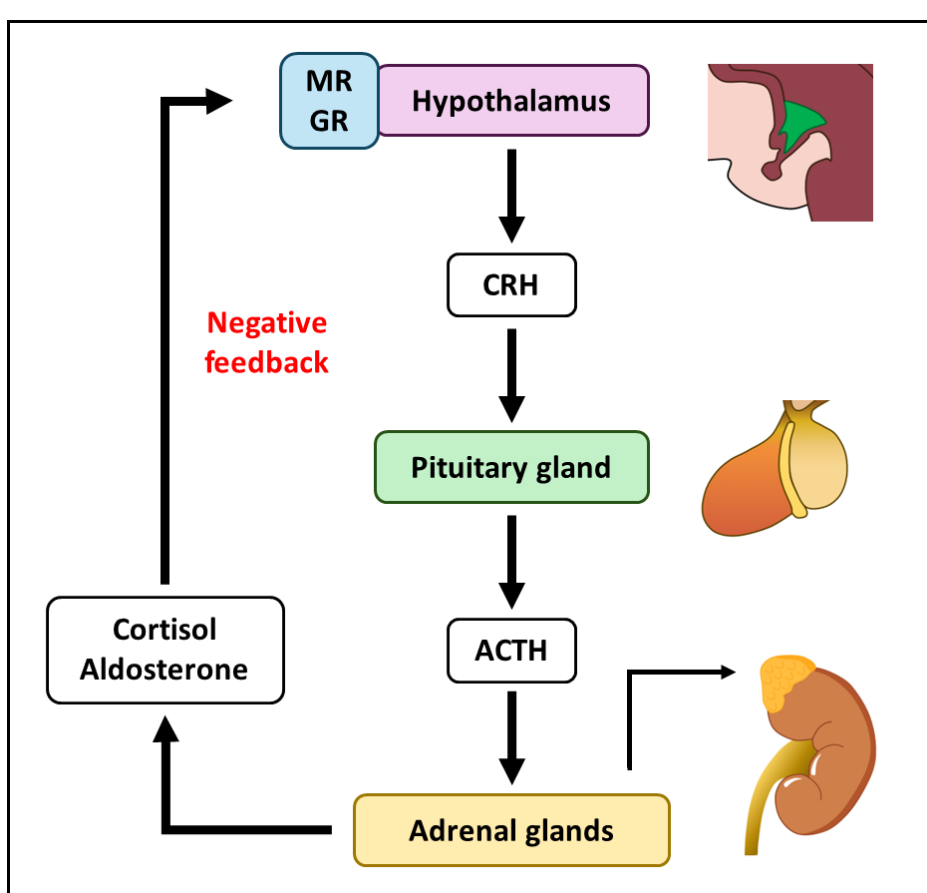
Hypothalamic–pituitary–adrenal (HPA) axis components and regulations. The delivery of corticotropin releasing hormone (CRH) by the hypothalamus to the pituitary gland induces the secretion of adrenocorticotropic hormone (ACTH). ACTH leads to the adrenal glands producing cortisol and aldosterone, which mediates negative feedback to the hypothalamus through the mineralocorticoid receptor (MR) and the glucocorticoid receptor (GR).

The cortisol induced by stress is largely regulated by the HPA axis. The HPA axis is primarily responsible for the synthesis and secretion of cortisol in the adrenal glands. The anterior pituitary or adenohypophysis produces and secretes adrenocorticotropic hormone (ACTH) in response to corticotropin-releasing hormone (CRH), released by the PVN of the hypothalamus [76]. Next, the adrenal glands produce and secrete cortisol in response to ACTH [77]. In turn, blood cortisol levels close the cycle by feeding back to the CRH-producing neurons, which stop releasing CRH when cortisol levels are elevated [78]. Therefore, glucocorticoids exert negative feedback on the hypothalamus, reducing the stress levels (Figure 1). Some regions of the hippocampus influence the HPA axis and susceptibility to stress [79]. 

The HPA axis is characterized by the generation of rhythms. Cortisol follows two types of rhythms, ultradian and circadian rhythms. The circadian rhythm is strongly associated with light–dark cycles and food availability [69]. Ultradian rhythms are due to pulsatile ACTH secretion [80]. In the hippocampus, the coordinated action of MR and GR activation with the innervation of CRH-producing neurons of the hypothalamus leads to a decrease in ACTH synthesis and secretion and, therefore, to a reduction in cortisol release [81]. Activation of the HPA axis during stress produces rapid increases in cortisol or corticosterone [76]. Both bind the MR with high affinity and subsequently occupy the GR. Binding to the MR produces excitation of hippocampal neurons in the first few minutes [69]. Thus, activation of the MR constitutes the initial response to stress, acting on the genome and maintaining the stability and integrity of the system. After these initial moments, the GRs become progressively engaged, initiating the end of the response to the stressor by triggering slow mechanisms at the genomic level. In these cases, GR expression levels correlate with sensitivity to negative situations. Indeed, an excess of stress or glucocorticoids during fetal development may be responsible for an increased risk of cardiovascular, metabolic, or neuroendocrine disorders in adulthood [82,83]. 

In summary, the MR not only has a role in the kidney and electrolyte homeostasis, but it is also expressed in the CNS, and it is very important in the HPA axis and the stress response. 

## 3. Sleep

Sleep is a highly conserved process in the animal kingdom. Although the exact function of sleep is not very clear, it is known to be present even in animals without a CNS [84]. Since animals are helpless while they sleep, and do not eat or reproduce, it is difficult to explain why they sleep. The theory of inactivity states that this is the very purpose of sleep, to keep the animal inactive once it has accomplished all these tasks (eating or reproducing); in fact, this seems to be a safer activity than wandering, exposing itself to predators and wasting energy [85]. There are other theories: the theory of energy conservation, restorative theory, and the theory of brain plasticity. The first theory states that sleep is a process to reduce energy expenditure during times when it is more difficult to hunt or gather food. Restorative theory claims that sleep time is a time for the organism to repair and replenish cellular components damaged or depleted during waking time. Finally, the theory of brain plasticity asserts that the process of brain growth and reorganization occurs when the organism is asleep [86]. Sleep may fulfill several aspects of these theories rather than only one of them. Despite the great unknowns when talking about sleep function, it is currently accepted that sleep is important for memory consolidation [87]; regulation of the immune system, e.g., antigen-specific antibody and T-helper cell responses have been shown to be enhanced in nighttime sleep compared to wakefulness [88,89]; and regulation of metabolism, directly affecting glucose tolerance as well as the appetite [90].

Basically, the sleep process can be divided into two phases, non-rapid eye movement (NREM) sleep and rapid eye movement (REM) sleep. In turn, NREM is divided into three stages: (1) stage 1 NREM sleep, which lasts between 1 and 7 min, in which sleep is very light and the individual can wake up very easily; (2) stage 2 NREM sleep, which lasts between 10 and 25 min, in which sleep much deeper than in stage 1, but the individual can still be woken up with a strong stimulus; and (3) stage 3 NREM sleep, lasting 20–40 min, in which sleep is deep sleep, also known as Slow-Wave Sleep (SWS), a time when memory consolidation [87] and immunity boosting activities take place [88,89] and when the organism is less metabolically active [90]. REM sleep is the dreaming phase of sleep. Voluntary muscle contraction stops except for the eyes, producing the eye movement characteristic of this phase. Brain activity during this phase is similar to wakefulness, and the body does not regulate temperature adequately. As people age, sleep becomes fragmented and the efficiency of sleep is lower [91]. Nighttime sleep is composed of 4–5 cycles of these stages. As the night progresses, the duration of the different stages changes, with more time in stage 2 NREM sleep and REM sleep [92,93].

There are several brain structures involved in sleep. The basal forebrain can promote both sleep and wakefulness. Cholinergic neurotransmission is active during wakefulness and REM sleep [94]. The reticular activating system maintains wakefulness by sending excitatory signals to the basal forebrain, thalamus, and hypothalamus [94]. The thalamus regulates sleep-related oscillatory activity and has been proposed to integrate central and decentral sleep signaling to produce a global signal [95]. In the hypothalamus, the lateral hypothalamic area consists of different types of neurons that can promote wakefulness as well as SWS and REM sleep [94,96].

In 1982, Borbely postulated that the sleep process is regulated by the hypothalamus by two mechanisms: the sleep–wake pattern (S-process) and the circadian clock (C-process). The interaction between these two factors determines the amount of time that we spend asleep [97]. Later, a third actor, the location factor (Z-process), was introduced, because it had been shown that electroencephalogram slow-wave activity varies with different locations, adding a geographical variable [98]. In this review, we focus on the circadian clock.

### 3.1. The Circadian Clock

The existence of circadian rhythms was discovered in 1937 in *Drosophila melanogaster* [99]. Using this same model, in 1971, Konopka and Benzer found a genetic base underlying this circadian process [100]. Since then, it has been shown that an internal clock exists in almost all living organisms, from animals and plants to bacteria and archaeobacteria [101]. This internal clock can synchronize with some environmental signals. This ability is called “entrainment”. These signals used by the clock for entrainment are called zeitgebers (time-givers); the most important zeitgebers are the dark–light pattern, the metabolic homeostasis, and the need for rest [102,103]. 

In mammals, the circadian clock is controlled by the hypothalamic suprachiasmatic nucleus (SCN), a small network of neurons capable of producing autonomous circadian rhythms [104]. There are peripheral circadian oscillators that operate independently but are entrained by the SCN. Nevertheless, they are entrained by other environmental cues and provide an independent circadian functioning of the tissue where they are expressed [102]. Light is the strongest known zeitgeber for the central clock [102]. Neurons from the hypothalamic SCN receive light input through the retinohypothalamic tract, which constitutes the photic stimulus input for entrainment of the internal clock with the light–dark pattern [102]. There are other signals that these peripheral clocks can use for entrainment: food availability, body temperature, or circulating factors such as corticoids [103,105].

**Figure 2:**
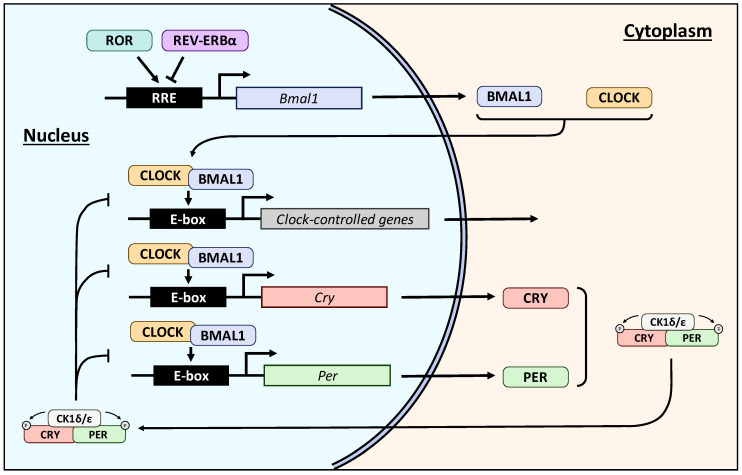
Circadian clock pathway. CLOCK: Circadian locomotor output cycles kaput, BMAL1: brain and muscle aryl hydrocarbon receptor nuclear-like 1, E-box: enhancer box, CRY: cryptochrome, PER: period circadian protein homolog, ROR: retinoid-related orphan receptor, REV-ERBα: reverse erythroblastosis virus α, CK1δ/ε: Casein Kinase 1 isoforms δ/ε, RRE: ROR response element.

The circadian clock is composed of a transcriptional machinery (cell autonomous transcription–translation feedback loop) that binds enhancer box (E-box) elements. Nearly 50% of genes have circadian regulation in mice and humans [106]. The circadian clock has regulatory arms. These include the positive regulator arm and the negative regulator arm. In the positive arm, Circadian locomotor output cycles kaput (CLOCK) and brain and muscle aryl hydrocarbon receptor nuclear-like 1 (BMAIL1) form a heterodimer binding the E-box motif in the promoter of the genes with circadian regulation. The genes *Cry* and *Per* also have E-box motifs in their promoters, increasing their transcription. Cryptochrome (CRY) and period circadian protein homolog (PER) belong to the negative arm of the circadian clock interfering with CLOCK and BMAL1 expression, inhibiting, therefore, their own transcription. The NR retinoid-related orphan (ROR) receptor and reverse erythroblastosis virus α (REV-ERBα) are part of the positive arm and the negative arm of the circadian machinery, respectively. The former activates and the latter inhibits the transcription of BMAL1 [107]. The protein casein kinase (CK)-1δ/ε is an important regulator of the cell cycle. CK-1δ/ε controls the PER function by phosphorylation [108,109] (Figure 2).

It has recently been shown in *D. melanogaster* that chromatin accessibility also follows a 24-h oscillation and could play an important role in the maintenance of the internal clock [109]. The proper functioning of the circadian clock depends on the activation of histone acetyl/methyl transferases [110]. It has even been observed that CLOCK, a member of the circadian core machinery, possesses histone acetyltransferase activity on its own [111].

Circadian regulation of the HPA axis has been described. There is a direct axonal connection between the SCN and the PVN. Furthermore, circadian secretion of CRH leads to increased ACTH levels and decreased vasopressin release from the SCN, allowing the organism to reach a cortisol nadir [112]. Indeed, both endogenous MR agonists, aldosterone and cortisol, follow a circadian pattern of release [113,114,115]. Cortisol level varies throughout the day and is also regulated according to metabolic demands. There is a peak in cortisol after awakening, and it gradually decreases until sleep, reaching a nadir in the early night hours [113]. In the case of aldosterone, sleep increases the release of this hormone and this correlates with plasma renin activity but not with cortisol. After awakening, aldosterone levels experience a large increase associated with increased adrenal gland activity and correlated with cortisol levels [114]. Thus, aldosterone is controlled by both sleep and the circadian clock [115]. The circadian clock is a strong determinant of the wake/sleep cycle, and its dysregulation could be the cause of difficulties in initiating and maintaining sleep or insomnia [116].

### 3.2. Role of the Mineralocorticoid Receptor in Sleep

Direct regulation of REM and SWS by cortisol has been described. Cortisol reduces REM, via GR activation, and increases SWS, via MR activation [1]. In fact, it seems that the activation of the MR is necessary to enter SWS and consolidate memory. However, when cortisol levels are elevated, both the MR and the GR are engaged, and this activation of the GR impairs SWS entry and memory consolidation [117]. A study in healthy young men shows that blocking the MR impairs memory consolidation, whereas blocking the GR improves it [118]. In the early evening, cortisol levels are lowest, allowing an MR occupancy rate of about 50–70% without GR occupancy [117]. This MR/GR activation ratio has also been found to be crucial for the migration of naïve T-cells to lymph nodes, which contributes to immune memory formation [119]. 

MR activation is also responsible for the HPA axis inhibition observed during early nocturnal sleep in humans [120]. With aging, the hippocampus undergoes changes that end in a disinhibition of the HPA axis. Since cortisol reduces both SWS and REM, HPA axis activation could contribute to insomnia and sleep fragmentation observed with normal aging [91].

Dexamethasone, a treatment for pediatric acute lymphoblastic leukemia, has among its side effects emotional symptoms, behavioral problems, and sleep disturbances [121]. Dexamethasone is a highly selective GR agonist and has a very low affinity for the MR. This means that it can inhibit the HPA axis by reducing circulating cortisol, which reduces MR activation in aldosterone-free territories. The addition of hydrocortisone to dexamethasone treatment reduces both neuropsychological and sleep-associated side effects. These data suggest that the MR has an important role in mood, behavior, and sleep. Therefore, it is important to maintain MR activation in glucocorticoid therapy, such as dexamethasone treatment [121]. 

A special case is patients with ASD, who have a high incidence of sleep disorders [3,122]. An ASD study identified 69 genes associated with ASD and with a form of ASD caused by a mutation in the promoter of the *NR3C2* gene [123]. A zebrafish *NR3C2* KO model has confirmed features of ASD, including social behavior deficits and sleep disorders. Like ASD patients, *NR3C2* KO zebrafish exhibit problems in sleep initiation and maintenance [123]. 

MDD is a mental condition strongly associated with sleep disturbance and is related to chronic dysregulation of cortisol and the HPA axis. Patients with MDD have a shorter duration of SWS. In addition, aldosterone levels have also been proposed as a marker of MDD [2]. Low peripheral MR activation is a predictor of therapeutic refractoriness of MDD, so patients with MDD who have low plasma Na^+^ levels and low blood pressure may have a poor prognosis. These patients have a discrepancy between central and peripheral MR function, leading to increased aldosterone levels, which acts in the CNS producing anxiety-like symptoms, but has little effect in the periphery [124]. Recently, treatment with MRA has been suggested to treat anxiety-related behavior [125]. Treatment with *Glycyrrhiza glabra*, an 11β-HSD2 inhibitor, increases peripheral MR activation, reducing refractoriness in these patients [126]. However, MRAs have shown no clear beneficial effects in patients and, in addition, have side effects including an increased risk of type 2 diabetes mellitus [2]. In addition, aldosterone and primary aldosteronism have been found to produce depressive and anxious phenotypes, both of which are associated with poor sleep quality [127]. Finally, a particular subform of MDD is associated with hyperaldosteronism, and a possible role for drugs that interfere with the renin–angiotensin–aldosterone system (RAAS) seems to be an underused option that could provide a good outcome in the treatment of MDD, especially that associated with high aldosterone levels [128].

### 3.3. Sleep Quality in Human Diseases

Among the sleep disorders, insomnia is the most common. Insomnia has a prevalence of 10% in the general population and about 20% of the population suffers occasional symptoms of insomnia [129]. Individuals suffering from insomnia have poor sleep efficiency and sleep satisfaction. Symptoms include difficulty initiating sleep, difficulty maintaining sleep, and/or waking up early in the morning. To fully fit the diagnosis, these symptoms must occur at least three times per week for at least three months and must be accompanied by daytime disturbances [130].

Sleep disorders are associated with a risk of occupational injury due to a lack of alertness and fatigue [131]. Insomnia has been linked to many other health problems, but also prolonged sleep. For example, short sleep duration has been shown to increase the risk of cancer in Asians and prolonged sleep increases the risk of colorectal cancer [132]. A meta-analysis found that both short sleep (less than 7 h) and long sleep (more than 9 h) are associated with an increase in cardiovascular events and all-cause mortality [133]. In a study conducted in China involving 409,156 adults with no history of stroke, coronary heart disease, or insomnia, it was observed that individuals who slept between 7 and 8 h had the lowest risk, whereas those who slept too little or too much (≤5 h or ≥10 h) had a 10% and 12% higher risk of stroke and 23% and 22% higher risk of major coronary events, respectively [134]. These data are confirmed by another meta-analysis involving 528,653 people from 16 prospective studies, where it was observed that the lowest risk was found in individuals who slept 7 h, while an increase of 1 h of sleep compared to the baseline raised the risk of total stroke to 13%. However, in this case, no effect on risk was observed for individuals with little sleep [135]. Finally, another systematic review of 13 prospective studies with 122,501 subjects followed for 20 years concluded that insomnia increases the risk of developing or dying from cardiovascular disease [136]. 

Short sleep duration or chronic sleep deprivation is also known to increase the risk of type 2 diabetes mellitus [90], as well as the risk of developing MDD [137] or the risk of Alzheimer’s disease [138]. Insomnia has also been associated with a worse prognosis of congestive obstructive pulmonary disease [139]. Finally, insomnia symptoms among middle-aged and older adults were associated with increased all-cause mortality [140]. A recent study shows that individuals with moderate to severe obstructive sleep apnea (OSA) had an increased risk of CKD progression [141], although the reliability of studies showing an association between insomnia and somatic disorders is questionable due to problems with diagnosis and inconsistencies in the definition of insomnia and methodology [130]. Therefore, studies with a consensus methodology and an established definition of insomnia are needed to clarify the exact impact of insomnia on somatic disorders. 

## 4. Sleep and Kidney Function

### 4.1. The Circadian Clock Controls Kidney Function

The kidney is a vital organ for maintaining electrolyte homeostasis and water balance, thus playing an important role in the regulation of blood pressure, and is also responsible for the excretion of metabolic waste products. The kidney is the second peripheral organ with the highest circadian rhythm-dependent gene expression (almost 50%), including genes involved in blood pressure regulation and waste excretion [108,142]. Excretion of both Na^+^ and K^+^ follows a circadian pattern, being maximal during the day for humans and during the night for rodents [143]. 

Several mechanisms of Na^+^ balance are controlled by the circadian clock. For example, Na^+^/H^+^ antiporter 3 (NHE3) is regulated by direct binding of the BMAL1:CLOCK dimer to E-box motifs in the NH3 gene promoter [144] and also by PER1, one of the core members of the circadian clock. Pharmacological inhibition and small interfering RNA (siRNA) of PER1 decrease NHE3 levels [145]. In addition, the expression of Na^+^- glucose transporter 1 (SGLT1) is also reduced after pharmacological inhibition of PER1 [145]. PER1 deficiency causes lower ENaC expression. Thus, PER1 is involved in Na^+^ homeostasis in the kidney [108]. Indeed, *Per1* KO mice show increased natriuresis. Moreover, inhibition of CK1δ/ε prevents the entry of PER1 into the nucleus and decreases the levels of subunit α of ENaC (αENaC) [108,146]. On the other hand, the decrease in PER1 levels is accompanied by a decrease in FXYD domain-containing ion transport regulator 5, also named dysadherin in humans or RIC in mice, which controls Na^+^/K^+^ ATPase activity, and an increase in ubiquitin-conjugating enzyme E2E3 (UBE2E3), caveolin-1 (CAV-1), and ET-1. UBE2E3 participates in the turnover of αENaC, reducing its presence in the membrane and marking it for degradation. CAV-1 and ET-1 contribute to the reduction in ENaC activity [108]. The NCC is also regulated by PER1. Pharmacological inhibition of PER1 decreased NCC expression and activity [147]. However, it appears that the regulation between aldosterone and PER1 is bidirectional, as PER1-deficient cells do not show the typical circadian pattern of increased aldosterone during the active phase. This may be due to a decrease in 3-(β)-hydroxysteroid dehydrogenase (3β-HSD) [108]. PER1, as well as the CRY1/2 protein, is part of the negative regulatory arm of the circadian clock. However, *Cry1/2* KO mice show opposite effects to those observed in *Per1* KO mice, which could be due to a differential effect of the components on distinct genes. Finally, *Cry1/2* KO mice exhibit elevated levels of 3β-HSD and aldosterone, as well as salt-sensitive hypertension [148]. 

**Figure 3:**
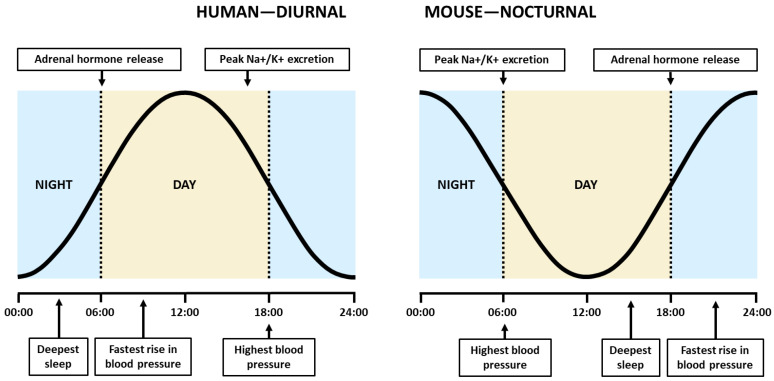
Kidney function circadian coupling. Representation of the circadian time (black line) with the peak of Na+/K+ excretion, the peak of blood pressure, and the peak of adrenal hormone release. Adrenal hormones: cortisol and aldosterone.

K^+^ balance is controlled by the circadian clock. The ratio of excretion between the lowest time of excretion to the highest one is 5:1 in humans with a normal K^+^ diet. According to this study, distal K^+^ uptake in the nephron follows a circadian pattern and is similar in all cells. There is a movement of intracellular K^+^ into the extracellular compartment in the morning and intercalated cells produce a similar movement of K^+^ into the distal nephron fluid, thus increasing K^+^ excretion. The opposite occurs in the late afternoon [149]. In line with this, there is a circadian variation in the expression of H^+^/K^+^ ATPase type 2, the pump responsible for K^+^ resorption from the distal nephron [150]. Thanks to the work of Firsov et al., in which they microdissected the DCT, connecting segment, and CCD segments from mice every 4 h for a 24 h period, circadian-regulated K^+^ channels are known. Among the regulated genes are the major elements of distal tubule K^+^ transport, such as the catalytic subunits of H^+^/K^+^ ATPase (*Atp4a* and *Atp12a*), several subunits of Na^+^/K^+^ ATPase (*Atp1a1*, Atp1a2 and Atp1b2), and the β subunit of the BK channel (*Kcnmb1*). Other genes, such as several voltage-gated K^+^ channels and inward rectifiers, including the Kir1.3 inward rectifier (*Kcnj15*), were also found [143,151]. Finally, Clock KO mice exhibit aberrant K^+^ excretion, showing again the regulation of K^+^ fluxes by the circadian clock [143] (Figure 3). 

The Na^+^ and K^+^ excretion peaks overlap, which is counterintuitive. The peak in Na^+^ excretion is thought to be due to a decrease in Na^+^ reabsorption, while the peak in K^+^ is due to an increase in K^+^ secretion. Indeed, the diuretic drug amiloride seems to blunt the peak in K^+^ excretion while increasing that in sodium Na^+^. Therefore, ENaC-ROMK regulation may play a role in this effect [143]. ROMK shows a circadian variation of 30% during the zeitgeber time 20, coinciding with the second part of the activity period and the peak of K^+^ excretion in mice [151] (Figure 3). 

Water uptake is also affected by the circadian clock. The type 2 vasopressin receptor, as well as aquaporin-2 and aquaporin-4, responsible for the water resorption in the collecting duct, peak in expression in the middle of the active phase. *Clock* KO mice show a phenotype similar to diabetes insipidus-like, a condition in which vasopressin signaling is impaired and water reabsorption capacity in the distal nephron is weakened [151,152]. 

Therefore, the kidney is tightly regulated by the circadian machinery.

### 4.2. Sleep Quality in Chronic Kidney Disease Patients

The role of sleep disorders, including insomnia, in the pathophysiology of several diseases has been discussed. However, this association is bidirectional, and some diseases, including ASD [3,122], MDD [121], Alzheimer’s disease [91], and cancer [153] have a high incidence of sleep disorders. In this line, CKD increases the prevalence of sleep disorders. Besides problems related to renal function such as anemia, bone disorder, loss of electrolyte balance, and accumulation of waste products, patients with CKD suffer other types of central alterations such as depression, anxiety, and insomnia or poor sleep quality [4,154,155]. Indeed, sleep disorders and insomnia constitute one of the comorbidities of CKD that impair the quality of life of patients with kidney disease. The three most frequent sleep disorders are sleep apnea, RLS, and insomnia [156]. Sleep disorders have been associated with both CKD and non-CKD-related renal disorders, including RLS, parasomnias, circadian sleep–wake disorders, hypersomnolence, central sleep apnea (CSA), OSA, and hypoventilation [156]. 

The incidence of these disorders in the general population is different from that in patients with CKD. For example, sleep apnea, which produces a worse evolution of kidney function, has two different origins: OSA and CSA. In both cases, the incidence is higher in CKD patients than in the general population, 25–57% vs. 10–49% for OSA and 10% versus 1% for CSA, respectively [4,141]. The prevalence of RLS in the general population is 5%, while in CKD patients it grows as the renal disease progresses and increases to 15–30%. CKD patients with RLS are three times more likely to have insomnia than those without RLS [4,157]. Interestingly, this association may be bidirectional, and sleep disorders may be responsible for an increased prevalence of RLS [158]. The prevalence of insomnia in CKD patients ranges from 38 to 70%, whereas in the general population, the values are between 10 and 20% [4]. Despite not modifying the progression of renal disease, insomnia increases the risk of cardio-cerebrovascular disease and all-cause mortality risk in patients with end-stage renal disease [159,160].

In a study conducted in patients diagnosed with CKD, the prevalence of poor sleep quality and insomnia was 59% and 48% for CKD patients without kidney replacement therapy (KRT), 68% and 46% for patients on hemodialysis, 67% and 61% for patients on peritoneal dialysis, and 46% and 26% for renal transplant patients. In addition, age seemed to increase the prevalence of insomnia, being higher in patients over 50 years than in younger patients [5].

As mentioned above, patients on KRT show a higher prevalence of insomnia, especially those on peritoneal dialysis [5]. Interestingly, the time of dialysis seems to have an impact on sleep disturbances. It has been observed that patients on evening dialysis show longer nocturnal sleep time, less daytime sleepiness, and need less hypnotic medication [161]. On the other hand, nocturnal hemodialysis improves sleep apnea symptoms [157]. Within patients on KRT, renal transplantation seems to have a beneficial impact on sleep quality and insomnia, since several studies showed an improvement in sleep quality in transplanted patients compared with when they were receiving hemodialysis [5,162,163]. However, another study with patients undergoing renal transplantation did not show an improvement in either sleep quality or nocturnal blood pressure dipping, but it did improve daytime sleepiness [163]. Baker et al. reported an improvement in sleep quality after transplantation in male patients but not in female patients [164]. Nevertheless, sleep quality remains a big problem even after transplantation, being one of the factors impairing the quality of life of these patients [165]. In addition, a Cochrane systematic review published in 2015 shows how different interventions have little to no effect in the management of sleep quality in CKD patients and highlights the need to address this pitfall in CKD symptom handling [166]. In short, more studies are needed to establish how to improve sleep quality in CKD patients.

## 5. Role of the Mineralocorticoid Receptor in Sleep Disorders in CKD

Although sleep quality and the prevalence of sleep disturbances are important in patients with CKD, unfortunately, sleep disturbances are poorly controlled in patients with CKD [166]. To the best of our knowledge, there is no existing literature that evaluates the use of the MR as a target to treat sleep problems in the context of CKD. However, below, we discuss the role of the MR in CKD and hypothesize whether the impaired MR function observed in patients with CKD could be responsible for the impaired sleep quality described in these individuals.

There is a much literature describing the role of the MR in the pathophysiology of CKD. At the end of the 20th century, MRAs began to be proposed as a treatment for CKD. The beneficial effects of spironolactone, the first MRAs developed, in cardiovascular and renal disease, have been described [167]. The side effects caused by spironolactone, including progestational and anti-androgenic effects such as gynecomastia, abnormal menstrual cycles, and impotence, led to the development of eplerenone, another steroidal MRA [167]. Since then, many studies have reported the benefits of MRA therapy in patients with CKD for both renal [6,168] and CKD-associated comorbidities such as vascular calcification [169], insulin resistance [170], left ventricular mass and function, blood pressure, and vascular stiffness [171]. All studies to date have found hyperkalemia to be a worrisome effect of treatment in patients with CKD [6]. The AMBER trial studied one of the strategies adopted to overcome hyperkalemia after MRA treatment: the use of drugs such as patiromer that prevent K^+^ absorption in the gastrointestinal tract, which allows prolonged treatment of CKD patients on an MRA [172]. Preclinical studies have been conducted demonstrating the effect of steroidal MRAs [170,173] and modern non-steroidal MRAs, which have a lower incidence of side effects [174,175]. Finerenone, a non-steroidal MRA, has been studied as a treatment in patients with CKD and type 2 diabetes mellitus for renal function and cardiovascular events [7] and in the new onset of heart failure [8].

Furthermore, as mentioned, the MR is involved in electrolyte homeostasis [10,58], so an imbalance in these electrolytes, involved in renal physiology, could alter circadian signaling and, therefore, CKD-associated sleep disturbances. Indeed, a high-salt diet advances peripheral circadian rhythms by 3 h in mouse livers, kidneys, and lungs [176] and delays them by 5.5 h in rat kidneys [177]. Along the same line, a reversal of circadian K^+^ excretion has been observed, with a 39% increase in nocturnal urinary K^+^ levels in patients with CKD [143]. Moreover, an imbalance in the Na^+^ excretion rhythm accompanied by an altered nocturnal blood pressure dipping has been described in CKD [178].

CKD also increases plasma aldosterone and cortisol plasma because of impaired urinary clearance [179,180]. In CKD, diurnal cortisol levels remain similar to healthy levels, but the rate of decline is lessened and nadir levels are not reached [180]. This cortisol can regulate the HPA axis, prevent the cortisol nadir needed to enter the SWS phase, and activate the renal MR upon a decrease in 11β-HSD2 activity, which is known to occur in CKD [181]. High levels of aldosterone are common in CKD [179]. High aldosterone levels correlate with poor prognosis and increase cardiovascular risk [182]. Aldosterone can also activate the central MR, altering MR occupancy and the GR/MR activation rate, which are known to be important for entering the SWS phase [117,118]. 

### 5.1. MR-Related Mechanisms Able to Trigger Sleep Disturbances in CKD

Although, to our knowledge, MR-related mechanisms capable of triggering sleep disturbances in CKD have not been described so far, here we propose two possible mechanisms by which CKD could lead to sleep disturbances. 

#### 5.1.1. Circadian Clock Disruption

**Figure 4:**
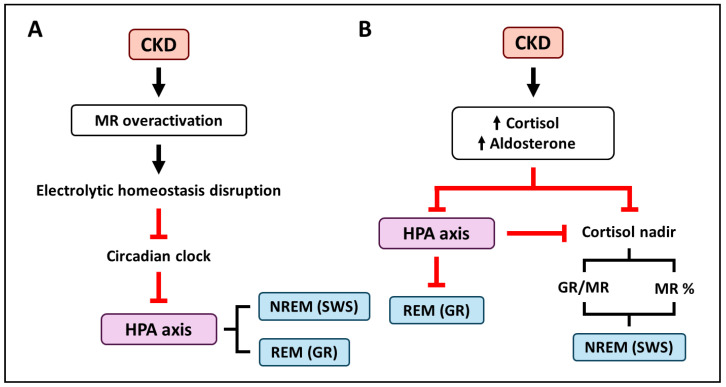
Potential mechanisms by which chronic kidney disease (CKD)-associated mineralocorticoid receptor (MR) dysregulation may lead to sleep disturbances. (**A**) The electrolytic imbalance observed in CKD through the overactivation of the MR could disrupt the circadian clock, which controls sleep and the hypothalamic-pituitary-adrenal (HPA) axis. Then, the altered HPA axis could dysregulate non-REM sleep phase (NREM) and rapid eye movement sleep phase (REM). (**B**) Hyperaldosteronemia and hypercortisolemia associated with CKD could lead to sleep disturbances in two ways. On one hand, through the HPA dysregulation and the consequent over occupancy of the glucocorticoid receptor (GR). On the other hand, increased levels of cortisol and aldosterone, as well as HPA axis dysregulation, lead to overactivation of the central GR and MR and entrance into the SWS phase.SWS: Slow-Wave Sleep.

CKD is accompanied by dysregulation of the MR, which is involved in electrolyte balance [10,58]. This electrolyte imbalance can dysregulate the circadian clock [143,176,177,178], which is a strong determinant of the wake/sleep cycle. Dysregulation of the circadian clock is one of the causes of difficulties in initiating and maintaining sleep or insomnia [116], possibly through the HPA axis or the regulation of other substances such as melatonin [183] (Figure 4A).

#### 5.1.2. High Levels of MR Agonist

Both MR ligands, aldosterone and cortisol, are increased in patients with CKD. An elevated aldosterone level in CKD patients is an independent risk factor for CKD progression [181]. In addition, the RAAS has also been associated with depression, which is often accompanied by insomnia [128]. On the other hand, plasma cortisol levels increase in CKD, producing some Cushing’s syndrome-like symptoms, such as depression and cognitive impairment, due to dysregulation of the HPA axis [182]. As mentioned above, cortisol can promote SWS entry through MR activation and reduce REM via GR activation [1]. Therefore, the elevated cortisol levels observed in CKD may alter sleep in two ways: through overactivation of the MR and through alteration of the GR/MR activation ratio [113,117,118,119] (Figure 4B).

### 5.2. Putative Therapeutic Interventions

#### 5.2.1. Insomnia-Related Regulatory Protein-Based Therapeutic Intervention

Once the relationship between the MR and CKD sleep disturbances is established, other molecular mediators that have been linked to sleep disturbances in CKD, such as orexin [184], could be studied in the context of MR activation. Orexin has been implicated in sleep problems [185] and also follows a circadian pattern of expression [186]. However, the relationship between MR activation and orexin has not been studied. 

#### 5.2.2. RAAS-Based Therapeutic Interventions

CKD therapy often includes drugs that interfere with the RAAS. It is not easy to speculate whether these drugs produce a good or a bad effect on the sleep of CKD patients. In fact, RAAS-based therapy could improve or worsen sleep quality due to its effect on aldosterone levels or MR function. In the case of MRAs, the classical steroidal components cross the blood–brain barrier, producing CNS effects. It has been seen that a blockade of the central MR can lead to an increased risk of depression and sleep disturbances. However, modern non-steroidal MRAs do not reach the central MR. On the other hand, a discrepancy between central MR activation and peripheral MR activation could occur, leading to an increased risk of sleep disturbances and insomnia [124,126].

Therefore, these drugs may not be the best choice either. Perhaps a partial agonist could be the best option, leading to antagonism of the natural ligands but preserving a part of the function of the receptor. Another aspect to consider is that, since the circadian clock is involved, the intervention is as important as the timing of the intervention, so perhaps chronotherapy should be considered to treat CKD patients, not only in KRT, but also in drug administration. For example, preventing MR activation at night could preserve the nighttime blood pressure dipping, which is important to maintain the functioning of the circadian clock.

Importantly, sleep disorder observational studies should be performed in clinical trials with CKD patients, annotating the quality of the sleep and the effect of the treatment administered on these disturbances. The timing of the doses and co-factors such as the use of stimulants or previous occurrences of insomnia, anxiety, or depression should be considered. 

## 6. Conclusions

It is demonstrated that the MR plays an important role in the physiology and pathophysiology of sleep, especially in the SWS phase and associated functions such as memory consolidation and immune system boosting. Partial occupancy of the MR and low occupancy of the GR by cortisol appears to be important for sleep maintenance, so the GR/MR ratio should be considered. Finally, the discrepancy between central and peripheral MR function as well as low peripheral MR function could be responsible for poor sleep quality.

### Further Research

Sleep disorders are a major problem in the quality of life of patients with CKD, so more attention should be paid to this symptom. 

On the one hand, more preclinical research is needed to elucidate the possible role of the MR in CKD-related sleep disorders. Although there are several animal models for insomnia, such as caffeine- or stress-induced insomnia in rodents [187,188], probably none of them would be a good choice, since the target is CKD-related insomnia. Therefore, the spontaneously hypertensive rat model described here, which exhibits sleep disturbances, may be a better choice [189]. Nevertheless, all of these preclinical models may be useful to demonstrate whether sleep disturbances further deteriorate the kidney by accelerating the progression of CKD and whether MRA could prevent this kidney deterioration. To conduct a rapid study of CKD-related sleep disorders, it would be necessary to monitor CKD animals by high-definition video recording with artificial intelligence support [190,191]. 

On the other hand, studies are needed to analyze the incidence of sleep disorders in CKD patients treated or not with drugs that interfere with the RAAS. This would allow a more detailed analysis of the role of the MR in these CKD-associated sleep disorders. Another interesting scenario would be to separate patients receiving steroid MRAs from those receiving non-steroidal MRAs, which would provide a great perspective on the appropriateness of these treatments. 

In short, advances in the knowledge of this comorbidity could have a very positive impact on the quality of life of patients with CKD and perhaps on the progression of kidney disease.

## Figures and Tables

**Table 1:** Natural and synthetic compounds and their ability to bind the mineralocorticoid receptor (MR)/glucocorticoid receptor (GR).

Compound Name	Binding Receptor	Origin	References
11-Deoxycorticosterone	MR	Natural	[26]
Aldosterone	MR	Natural	[27]
Corticosterone	MR/GR	Natural	[28]
Cortisol	MR/GR	Natural	[29]
Cortisone	MR/GR	Natural	[30]
Progesterone	MR	Natural	[31]
11-oxa-Cortisol	GR	Synthetic	[32]
11-oxa-Prednisolone	GR	Synthetic	[32]
19-Noraldosterone	MR	Synthetic	[33]
19-Nor-Desoxycorticosterone	MR	Synthetic	[34]
19-Nor-progesterone	MR	Synthetic	[35]
Beclomethasone	GR	Synthetic	[36]
Betamethasone	GR	Synthetic	[37]
Budesonide	GR	Synthetic	[38]
Deoxycorticosterone acetate	MR	Synthetic	[39]
Dexamethasone	GR	Synthetic	[40]
Dexamethasone oxetanone	GR	Synthetic	[32]
Eplerenone	MR	Synthetic	[41]
Finerenone	MR	Synthetic	[42]
Fludrocortisone	MR	Synthetic	[43]
Hydrocortisone	MR/GR	Synthetic	[44]
Prednisolone	MR	Synthetic	[45]
Prednisone	GR	Synthetic	[46]
Spironolactone	MR	Synthetic	[41]
Triamcinolone	GR	Synthetic	[47]
Vamorolone	GR	Synthetic	[48]
